# Reply to the letter: “Rethinking headache in juvenile systemic lupus erythematosus: the need for broader perpectives”

**DOI:** 10.1055/s-0046-1825764

**Published:** 2026-08-28

**Authors:** Bryan da Silva Marques Cajado, Renata Lopes Francisco de Andrade, Maria Angelina Carvalho Pereira, Marcelo de Melo Aragão, Maria Teresa Terreri

**Affiliations:** 1 Universidade Federal de São Paulo, Escola Paulista de Medicina, Departamento de Neurologia e Neurocirurgia, São Paulo SP, Brazil.; 2 Universidade Federal de São Paulo, Escola Paulista de Medicina, Departamento de Pediatria, São Paulo SP, Brazil.

We genuinely appreciate Christian Messina for the interest shown in our study and for his thoughtful feedback. The points he raised are excellent and add real value to the discussion on headache in juvenile SLE.

To address the questions about chronic headache causes: we decided not to use caffeine consumption, dehydration, or sleep deprivation as exclusion criteria simply because they are incredibly common among adolescents. If we had excluded everyone with these issues, we would have lost a huge part of our sample and hurt the study's representativeness. That said, we did analyze these factors as potential triggers. We also specifically looked for headaches related to sexual activity and substance use, but since those findings were negative, they were not included in the final text. We did not collect data on diving or barometric pressure, but we are more than happy to share the raw dataset if you wish to run further analyses.

Regarding the lack of brain magnetic resonance imaging (MRI), it is important to remember the context of our setting. The study in question was conducted in a public hospital within the Brazilian Unified Health System (SUS, from the Portuguese Sistema Único de Saúde), where access to routine MRI is limited without an acute clinical indication. Furthermore, given that our patients ranged from 5 to 18 years old, many would have required sedation. This adds significant cost and risk, especially for children with comorbidities like nephropathy who can be harmed by undergoing investigative procedures that require contrast agents or sedation. These are logistical and ethical constraints that are unfortunately quite common in pediatric research. Furthermore, if the history and physical examination are not suggestive of a secondary cause, no additional tests are required. 1

Thank you again for these valuable insights. We remain available for any further clarification.
